# Supplementary material for: Secretory Leukocyte Protease Inhibitor (SLPI)—A Novel Predictive Biomarker of Acute Kidney Injury after Cardiac Surgery: A Prospective Observational Study
Source: J Clin Med. 2019 Nov 9;8(11):1931. doi: 10.3390/jcm8111931 (PMC6912354; doi:10.3390/jcm8111931)
Supplement: Supplementary file 1 [file jcm-08-01931-s001.pdf]

Supplementary Materials

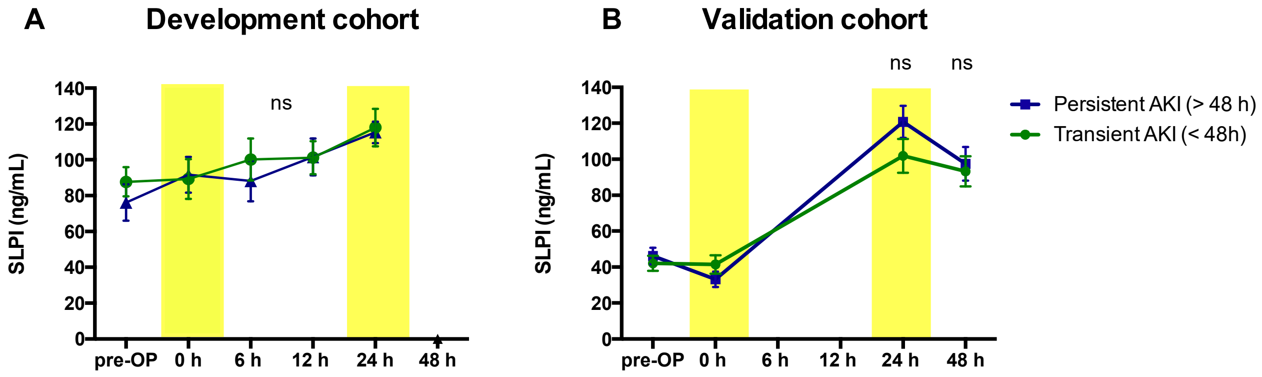

Figure S1. Comparison of serum SLPI in patients affected by transient AKI (< 48 hours) versus persistent AKI (> 48 hours) in the (A) development cohort and (B) validation cohort. SLPI, secretory leukocyte protease inhibitor. Data are means ± SEM; *P*-values versus other groups at the corresponding time point (24 h after surgery) (difference between groups) analyzed by Mann-Whitney *U* test.

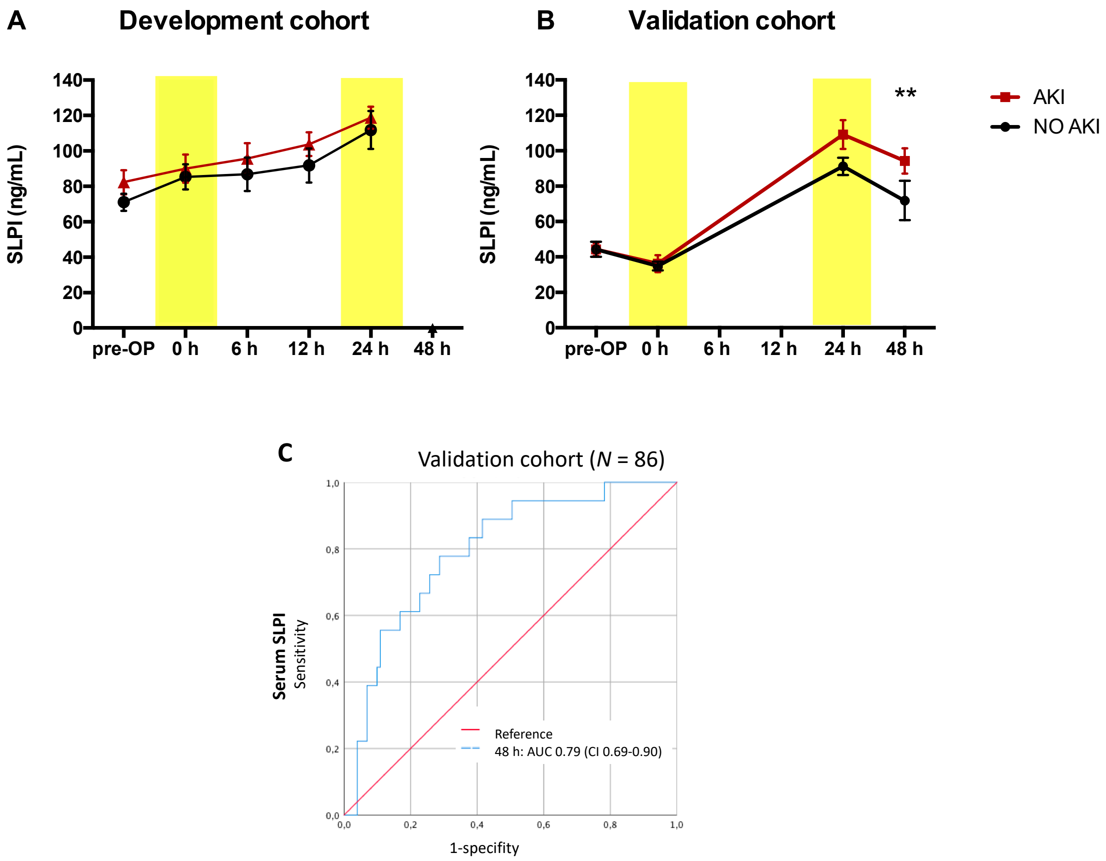

Figure S2. Diagnostic accuracy of serum SLPI in patients "at risk" as identified by Cleveland Clinic Foundation Score. Comparison of perioperative serum SLPI in patients identified as "at risk" by Cleveland Clinic Foundation Score ≥3. In these subgroups the incidence of AKI was 35% (13/37) in the development cohort (A) and 15% (13/86) in the validation cohort (B). (C) Receiver operating characteristic (ROC) curve of serum SLPI for the diagnosis of AKI 48 hours after surgery (validation cohort). AKI, acute kidney injury; AUC, area under the curve; CI, 95% confidence interval; SLPI, secretory leukocyte protease inhibitor. (A and B) $P < 0.01$ versus other groups at the corresponding time point analyzed by Mann-Whitney *U* test.

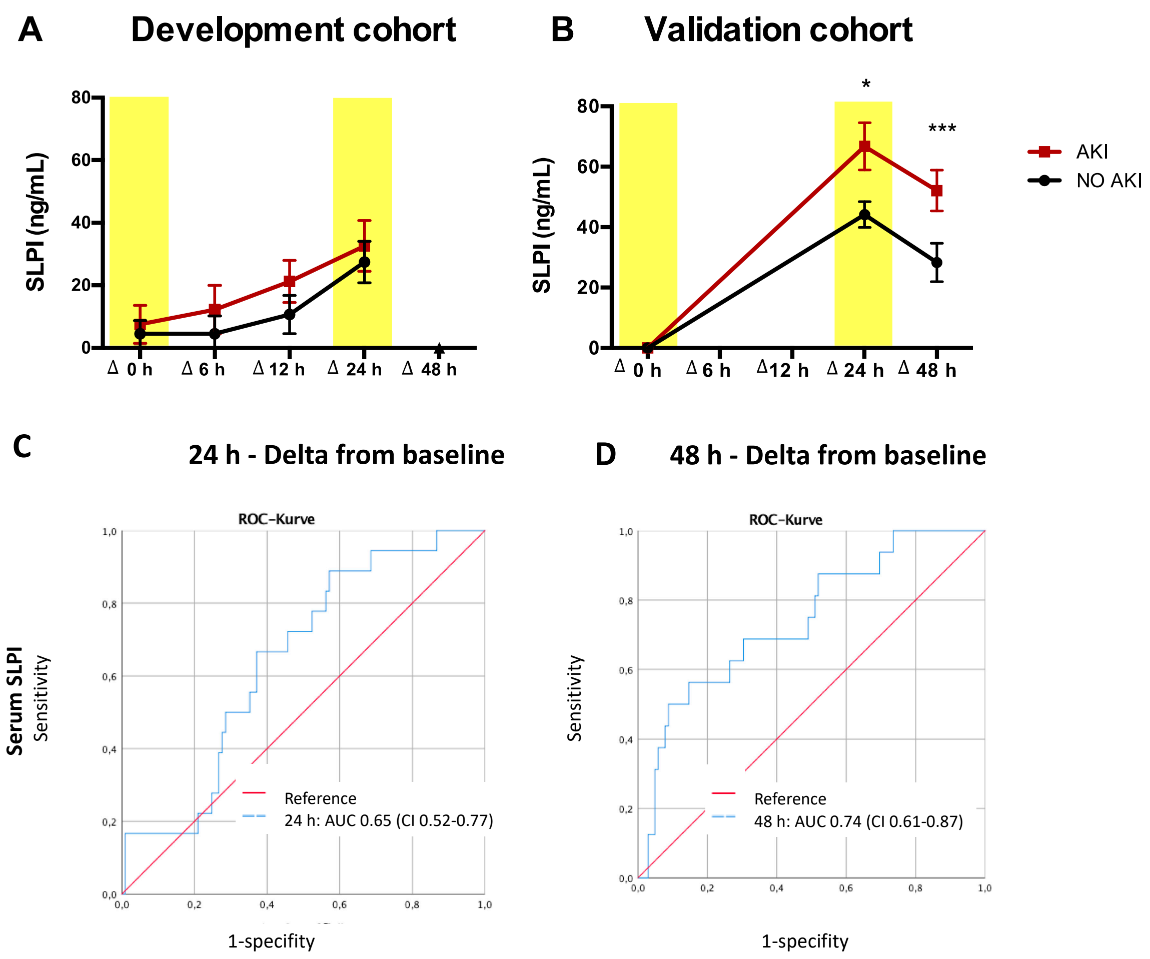

Figure S3. Association of absolute increase of serum SLPI from baseline (pre-OP, before surgery) with AKI. Comparison of the absolute increase of serum SLPI from baseline (delta from pre-OP) in AKI vs. non-AKI patients (A) in the development and (B) in the validation cohort. (C-D) Receiver operating characteristic (ROC) curve of serum SLPI for the diagnosis of AKI (C) 24 hours and (D) 48 hours after surgery (validation cohort). AKI, acute kidney injury; AUC, area under the curve; CI, 95% confidence interval; SLPI, secretory leukocyte protease inhibitor. (A and B) $P < 0.01$ versus other groups at the corresponding time point analyzed by Mann-Whitney U test.

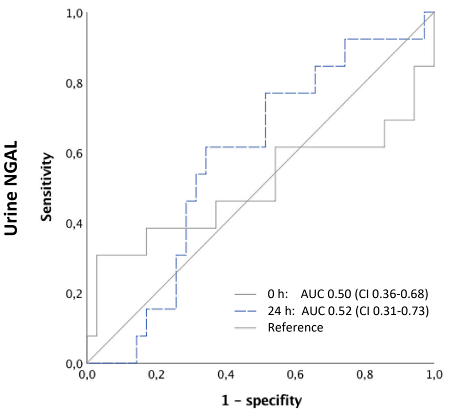

Figure S4. Receiver operating characteristic (ROC) curves of urine NGAL for the diagnosis of AKI. AKI, acute kidney injury; AUC, area under the curve; CI, 95% confidence interval; NGAL, neutrophil gelatinase-associated lipocalin.

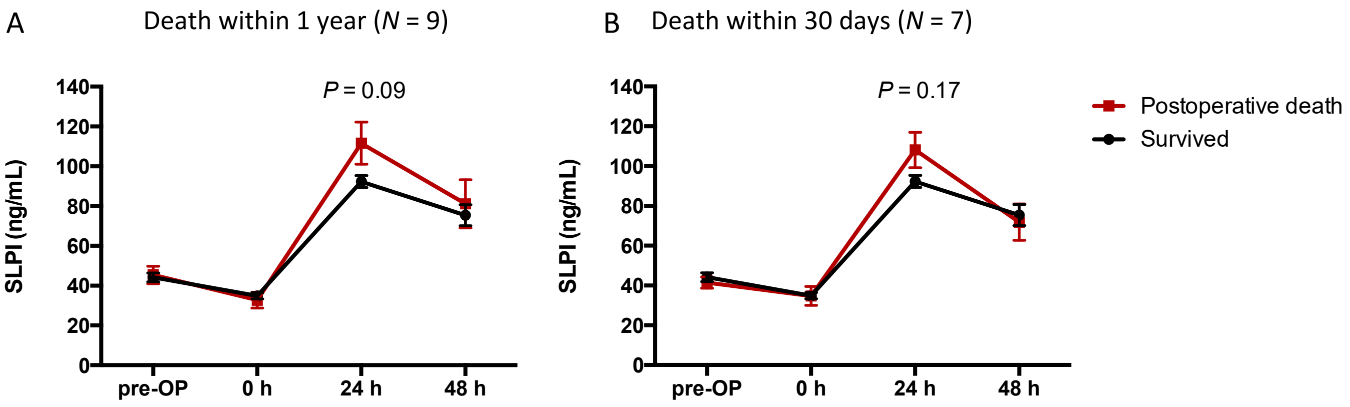

C

ICU length of stay	Number of patients
< 24 h	*N* = 74
24 h – 7 day	*N* = 65
> 7 days	*N* = 9

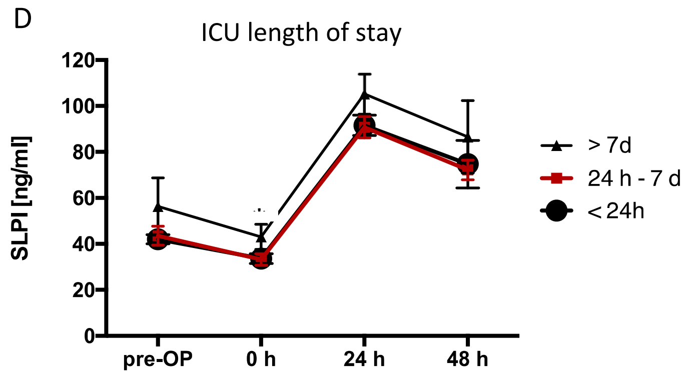

Figure S5. Association of perioperative serum SLPI with postoperative death and ICU length of stay. Perioperative kinetics of serum SLPI in patients who survived in the first year after surgery (*N* = 139) compared to patients (A) who died during in first year after surgery (*N* = 9) and (B) who died during the first 30 days after surgery (*N* = 7) (Validation cohort). (C) Overview of ICU length of stay. (D) Perioperative serum SLPI in relation to ICU length of stay. SLPI, secretory leukocyte protease inhibitor. Data are means ± SEM; (A and B) *P*-value versus other groups at the corresponding time point (difference between groups) analyzed by Mann-Whitney *U* test.
